# Structure-Controlled Polyetherimide Hollow Fibers for Biogas Purification

**DOI:** 10.3390/polym18080951

**Published:** 2026-04-13

**Authors:** Pavel Țiuleanu, Artem A. Atlaskin, Kirill A. Smorodin, Sergey S. Kryuchkov, Maria E. Atlaskina, Anton N. Petukhov, Andrey V. Vorotyntsev, Nikita S. Tsivkovsky, Alexander A. Sysoev, Ilya V. Vorotyntsev

**Affiliations:** 1 Laboratory of SMART Polymeric Materials and Technologies, Mendeleev University of Chemical Technology of Russia, 125047 Moscow, Russia; smorodin.k.a@muctr.ru (K.A.S.); kriuchkov.s.s@muctr.ru (S.S.K.); atlaskina.m.ev@muctr.ru (M.E.A.); tsivkovskii.n.s@muctr.ru (N.S.T.); sysoev.a.a@muctr.ru (A.A.S.); vorotyntsev.i.v@muctr.ru (I.V.V.); 2 Chemical Engineering Laboratory, Lobachevsky State University of Nizhni Novgorod, 603022 Nizhny Novgorod, Russia; antopetukhov@gmail.com (A.N.P.); an.vorotyntsev@gmail.com (A.V.V.)

**Keywords:** biogas upgrading, membrane, gas separation, carbon dioxide, hollow fiber, polyetherimide

## Abstract

Polyetherimide (Ultem-1000) hollow-fiber membranes were developed for biogas purification with emphasis on the relationship between spinning conditions, membrane morphology, gas transport properties, and module performance. Hollow fibers were prepared from dope solutions based on dimethylformamide (DMF) and N-methyl-2-pyrrolidone (NMP) at different conditions, followed by post-treatment with 1 and 3 wt.% silicone solution in *n*-heptane to reduce nonselective defects and improve selectivity toward the intrinsic behavior of dense PEI films. SEM analysis revealed that DMF-based fibers formed a more open, macrovoid-rich structure, whereas NMP-based fibers exhibited a more homogeneous sponge-like morphology with a better-defined selective layer. DMF-based fibers experienced faster demixing, which promoted macrovoid formation, increased pore connectivity of the substructure, lowered mass transfer resistance, and at the same time increased the probability of nonselective pathways and defect-related loss of selectivity. This structural evolution was reflected in gas transport properties: untreated DMF fibers showed high mixed-gas permeance but limited selectivity, while NMP fibers demonstrated lower permeance and selectivity values closer to those of the dense film. Silicone post-treatment significantly improved separation performance, with 3 wt.% coating being markedly more effective than 1 wt.% coating. The best compromise between permeance and selectivity was achieved for the DMF-based fibers treated with 3 wt.% silicone, which exhibited CO_2_ and H_2_S permeances of 39.4 and 47.12 GPU, respectively, together with selectivity values of 22.4, 26.8 and 20.2 for CO_2_/CH_4_, H_2_S/CH_4_ and CO_2_/N_2_. A membrane module containing 500 fibers was studied during the quasi-real biogas upgrading. With increasing stage-cut, the CH_4_ concentration in the retentate increased from ~74 to 96 mol.%, while CO_2_ decreased from ~21 to 2 mol.%. The results demonstrate that structure control combined with silicone post-treatment is an effective strategy for producing PEI hollow fibers suitable for simultaneous methane enrichment and removal of acid impurities from biogas.

## 1. Introduction

One of the most significant limiting factors for the development of civilization is energy generation [1]. Given that geological reserves of hydrocarbons are being rapidly depleted [2,3], including for energy generation, particular attention is being focused on alternative energy sources [4,5]. Although the development of energy sectors such as solar, wind, and nuclear power is highly valuable and beneficial, there is a vast infrastructure of energy facilities that operate by burning fossil carbon fuels. Therefore, developments based on biogas, the main component of which is methane, appear highly attractive [6,7,8,9]. However, in addition to methane, biogas also contains acid gases (primarily carbon dioxide and hydrogen sulfide), which reduce the quality of this fuel and inevitably lead to equipment corrosion. Consequently, there is a need for biogas purification.

Among traditional acid gas capture methods, pressure swing adsorption and amine scrubbing are particularly noteworthy [10,11,12]. However, these methods have significant drawbacks [13,14,15,16,17,18,19]. One of the main disadvantages is the high process cost, which can approach USD 111 per ton of captured or removed CO_2_, largely due to the substantial energy input required for solvent regeneration, often exceeding 3000 MJ per ton of captured gas [20,21]. Direct comparison with biogas upgrading technologies should be made cautiously, since biogas studies usually report costs per unit of upgraded biomethane rather than per ton of removed CO_2_. In the biogas upgrading literature, amine scrubbing is typically reported in the range of 0.20–0.35 USD/Nm^3^ biomethane, compared with 0.12–0.22 for pressure swing adsorption (PSA), 0.15–0.25 for water scrubbing, and 0.18–0.28 for membrane separation [22]. In addition, long-term operation is complicated by solvent degradation [23]. Exposure to elevated temperatures and oxygen-containing gas streams [24,25] promotes the formation of multiple byproducts, including ammonia, aldehydes, amides, and organic acids. Many of these compounds accumulate in the form of heat-stable salts that cannot be removed during conventional desorption. Their presence lowers the working capacity of the absorbent and negatively affects its physicochemical stability, which in turn enhances foaming, corrosion, and equipment damage [26]. Pressure swing adsorption is also difficult to scale, since the units can operate only in batch mode, and multiple units must be deployed to establish a continuous process. Sorbent regeneration in this case also requires significant energy consumption.

Membrane [27], hydrate-based [28] or hybrid technologies [29] represent an attractive and cutting-edge alternative. The separation technique alone allows for the extraction of acid gases from emission sources with greater economic efficiency, both in terms of capital and operating costs, without sacrificing gas recovery rate [22,30,31]. Another important advantage is the ease of scaling membrane plants due to their modularity, where the overall capacity may be changed by the number of membrane elements in parallel. Membrane materials themselves are also tunable through the combination of technological parameters during their production. Moreover, the only energy-demanding unit in the membrane gas separation route is the pressure changing device. There are no heat users or their producers; it is reagent free and because of that it is environmentally and industrially safe.

Nevertheless, one of the most significant challenges in membrane gas separation is the search for suitable membrane materials [32] and the combination of methods and technical solutions to produce the membrane suitable for a specific process. Numerous studies propose different membrane materials for biogas upgrading applications, such as mixed-matrix membranes [33,34,35], supported ionic liquid membranes [36,37,38] or graphene oxide membranes [39,40]. Nevertheless, these examples are lab-scale and can be hardly transferred to an industry-applicable form factor. The materials most easily scalable to industry standards are polymers. But also, geometry of the membrane plays a huge role in applicability. Hollow fibers are the most promising geometry to produce densely-packed membrane modules, so non-solvent or thermally induced phase separation (NIPS or TIPS) spinning is the go-to method for the production of engineeringly sound membranes [41,42]. As the fiber is obtained through the thin-hole spinneret, incorporation of solid particles into the dope solution is guaranteed to lead to process instability. Because of this, tuning of the membrane properties through the process of condition control or post-treatment is the most promising way to develop suitable material with scaling-up possibility. On the one hand, the membrane must possess mass transfer characteristics that meet the process requirements; on the other hand, it is essential that satisfactory materials can be produced easily on an industrial scale. For example, ceramic membranes demonstrate outstanding mass transfer characteristics [43,44], but even conceiving a method for mass-producing such membranes is a rather non-trivial task. One can also cite the example of polymer membranes [45,46], which possess favorable mass transfer characteristics but can only be produced in the form of flat films. At the same time, the most optimal and relevant form factor for gas separation membranes is hollow fibers.

The aim of this work was to develop polyetherimide (PEI, Ultem-1000) hollow-fiber membranes for biogas purification and to clarify how fabrication conditions affect membrane morphology, gas transport characteristics, and separation performance as the concentrations of selected polymers or ionic substances play a critical role. The present study is one of the steps in the comprehensive rigorous analysis of an optimization framework for PEI hollow-fiber production, and to achieve this aim, the study addressed several tasks: (i) determination of the intrinsic gas transport properties of dense PEI films as a reference for the polymer-selective behavior; (ii) fabrication of asymmetric hollow fibers using different solvents and spinning conditions in order to obtain membranes with tunable structure; (iii) establishment of structure–property relationships by correlating membrane morphology with mixed-gas permeance and selectivity; (iv) evaluation of silicone post-treatment as an approach for suppressing nonselective defects and improving separation performance; and (v) assessment of the applicability of the most promising PEI hollow fibers for biogas upgrading in a lab-scale membrane module under quasi-real mixed-gas conditions.

To accomplish these tasks, a combined material–process approach was applied. Dense PEI films and hollow fibers were prepared and systematically characterized using gas permeation measurements and SEM analysis of membrane cross-sections. The hollow fibers were fabricated from dope solutions based on N-methyl-2-pyrrolidone and dimethylformamide with variation of the air gap distance, which enabled controlled modification of the membrane structure from highly porous defect-prone morphologies to more homogeneous selective architectures. In addition, selected fibers were treated with dilute silicone solutions in *n*-heptane in order to seal surface imperfections and shift the transport behavior toward the intrinsic selectivity of dense PEI. It was expected that this strategy would make it possible to identify the optimal combination of spinning conditions and post-treatment, yielding PEI hollow fibers capable of methane enrichment together with efficient removal of carbon dioxide and hydrogen sulfide from biogas.

## 2. Materials and Methods

### 2.1. Materials

Polyetherimide (PEI) was selected as a well-established and readily tunable polymer for the fabrication of hollow-fiber membranes exhibiting stable and reproducible mass transfer characteristics. Commercial Ultem-1000 polyetherimide supplied by Sabic (Riyadh, Saudi Arabia) was used as the membrane-forming polymer. The spinning dope was prepared using dimethylformamide (DMF) and N-methyl-2-pyrrolidone (NMP) obtained from Ruskhim (Moscow, Russia), together with isopropanol (IPA) purchased from Khimmed (Moscow, Russia). In addition, reverse-osmosis water produced on-site, with a specific conductivity below 0.06 μS/cm, and hexane from Ruskhim (Moscow, Russia) were employed during the post-treatment of the prepared fibers. Silicone coating Sylgard 184 by Dow Corning (Midland, MI, USA) and n-heptane by Ruskhim (Moscow, Russia) were used to treat obtained hollow fibers.

The resulting membranes (films and asymmetric hollow fibers) were evaluated in permeation experiments using individual gases, including nitrogen (≥99.999%), oxygen (≥99.999%), hydrogen (≥99.995%), carbon dioxide (≥99.99%), methane (≥99.99%), and hydrogen sulfide (≥99.95%), supplied by NII KM LLC (Moscow, Russia), Voessen LLC (Moscow, Russia), and Firma Horst LLC (Moscow, Russia). Following the single-gas measurements, mixed-gas permeation behavior was investigated using a six-component model mixture composed of the same pure gases, namely methane, carbon dioxide, hydrogen sulfide, hydrogen, nitrogen, and oxygen, with the following molar composition: 68.3/27.13/0.56/1.92/0.97/1.12 mol.%.

### 2.2. Polyetherimide Film Preparation

Dense PEI films were obtained by casting polymer solutions prepared in chloroform. PEI dense films were fabricated from a 7 wt.% polymer solution in chloroform (analytical grade). For the evaluation of gas separation performance, films with a thickness of 50 ± 5 μm were produced by solution casting onto a cellophane substrate. The cast films were first dried under ambient conditions for 2–3 days and subsequently placed under vacuum (150 Pa) until a constant mass was reached.

In order to determine PEI diffusion coefficient with respect to hydrogen, additional films were prepared with increased thicknesses of 100 ± 7, 150 ± 7 and 300 ± 10 μm.

### 2.3. Asymmetric Hollow-Fiber Spinning

The non-solvent-induced phase separation (NIPS) technique was used to produce the asymmetric hollow-fiber membranes. Dope solution contained 29 wt.% of polymer in the case of NMP-based mixture and 27 wt.% in the case of DMF-based one. NMP and DMF contents in both solutions were the same and equal to 60 wt.%, while IPA concentrations were 11 and 13 wt.%. The dope composition was determined using a cloud-point technique based on gradual addition of the non-solvent to the polymer solution under continuous stirring. In this titration-like procedure, the endpoint was defined as the onset of irreversible partial coagulation, i.e., the appearance of a solid phase that no longer redissolved upon mixing. From a thermodynamic point of view, this state corresponds to approaching the precipitation boundary of the polymer–solvent–non-solvent system and therefore provides a practical estimate of the maximum non-solvent content compatible with a metastable homogeneous dope suitable for controlled phase inversion. The initial polymer content in the binary polymer–solvent system was chosen based on a variety of literary sources [47,48,49,50].

Therefore, the final dope solution compositions were

PEI/NMP/IPA—29/60/11 wt.%;

PEI/DMF/IPA—27/60/13 wt.%.

The spinning solution was prepared in several successive stages. First, NMP or DMF was introduced into the dope preparation vessel and heated to 50 °C. Under continuous stirring, PEI was gradually added in small portions in order to avoid polymer agglomeration and adhesion to the stirrer anchor. After complete polymer addition, the resulting intermediate mixture was maintained at the same temperature for at least 4 h. IPA was added to the solution. The final solution was stirred overnight to ensure homogenization and subsequently transferred to the spinneret reservoir, where it was left quiescent for no less than 8 h to allow entrapped gas bubbles to escape. The rheological behavior of the dope solution was studied using an Anton Paar MCR-702E rheometer equipped with a cone-and-plate measuring cell.

**Figure 1:**
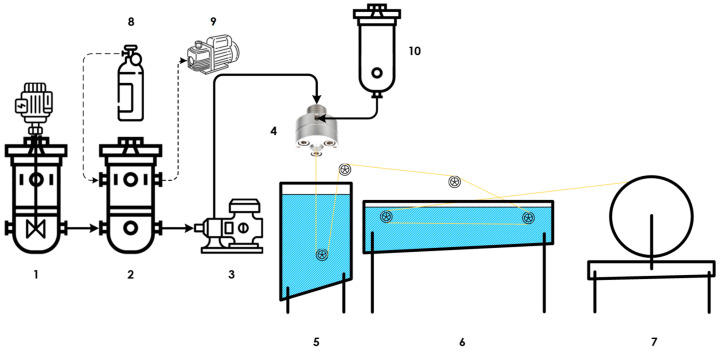
Hollow-fiber spinning setup principal scheme. Reproduced with permission from [51]. 2025 MDPI.

A schematic representation of the hollow-fiber spinning line is shown in Figure 1. The experimental setup included a dope preparation vessel (1), a spinneret reservoir (2), a gear pump (3), a spinneret (4), a coagulation bath (5), a washing bath (6), a winding unit (7), an argon cylinder (8), a diaphragm vacuum pump (9), and a bore liquid tank (10).

The polymer solution was prepared directly in a sealed dope vessel that had been preliminary purged with argon in order to exclude contact with atmospheric oxygen and moisture. Upon completion of solution preparation, the dope was transferred into the spinneret reservoir and kept without agitation for degassing. Both vessels, together with all connecting lines, were fitted with water jackets, enabling temperature control up to 95 °C. During spinning, the polymer solution was delivered by the gear pump to the outer channel of the spinneret, while the internal coagulant was supplied through the inner channel. The spinneret had an outer dope orifice diameter of 0.55 mm and an inner bore orifice diameter of 0.33 mm. After extrusion, the nascent hollow fiber entered the coagulation bath, where phase inversion and structure formation occurred, after which the fiber was washed and collected on the winding unit.

In all experiments, the polymer solution flow rate was fixed at 1.44 g min^−1^. Water was used as the bore fluid, also supplied at 1.12 g min^−1^. The temperatures of the dope solution and bore liquid were maintained at identical values, namely 25 °C or 50 °C, such that both streams were kept at the same temperature during spinning, whereas the coagulation bath temperature was kept at 25 °C. The take-up speed remained constant throughout the study at 17.5 m min^−1^. The only variable parameter was the air gap distance between the spinneret tip and the coagulation bath surface, which was adjusted from 8 to 16 cm in 4 cm increments. As a result, twelve hollow-fiber membrane batches with different morphologies and, accordingly, different mass transfer properties were produced.

To preserve the developed porous structure and minimize pore collapse, the freshly spun fibers were subjected to a sequential post-treatment procedure. Initially, the fibers were immersed in bidistilled water for 2 days. They were then transferred to isopropanol and kept there for another 2 days, followed by treatment in hexane for 8 h. After solvent exchange had been completed, the fibers were dried under vacuum for 12 h at a residual pressure of 15 kPa.

### 2.4. Membrane Mass Transfer Characterization

Here, the lab-scale hollow-fiber membrane modules were made using 1/4” stainless steel tubes and tees provided by Swagelok (Solon, OH, USA) using epoxy resin as a potting agent at the edges of the module. Each module contained 50 fibers randomly hand-picked from each obtained bundle. Firstly, the gas permeance tests were performed for PEI films, including single-gas permeation with further exposure to mixed gas (6-component quasi-real biogas). After the permeability, diffusion and sorption coefficients were determined for both cases, and the hollow fibers’ permeance was studied.

**Figure 2:**
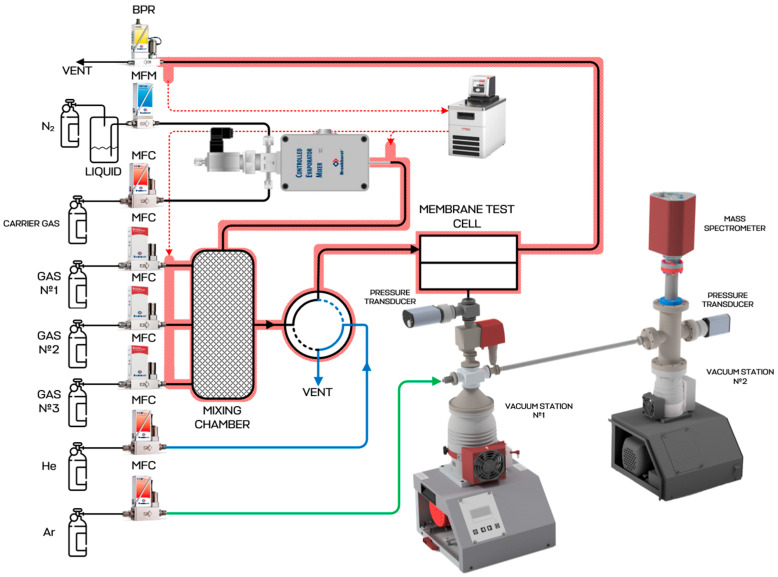
Schematic diagram of mass-spectrometer-coupled experimental unit for study of membrane mass transfer properties. Reproduced with permission from [51]. 2025 MDPI.

The membrane performance tests were conducted using a custom-built experimental rig fitted with a quadrupole mass spectrometer. A schematic representation of the system is shown in Figure 2. The gas-supply section of the setup was equipped with Bronkhorst FG-201 CV mass-flow controllers (Ruurlo, The Netherlands), which were used either for feeding pure gases or for preparing gas mixtures in real time by combining the required components in a mixing vessel. In addition, two Bronkhorst controllers, F-201 CV and F-201 CM (The Netherlands), were employed to deliver purge helium and argon as an internal standard to the test lines when these gases were not among the target permeants. Helium was introduced to flush the distribution lines from residual gases remaining after previous measurements and thus condition the unit before a new experiment. Switching of the membrane module feed side between the gas-mixture vessel and the helium line was accomplished using a pneumatically actuated two-way valve. On the retentate side, a Bronkhorst P702 CM pressure regulator (The Netherlands) was installed to maintain a constant pressure along the membrane module.

After passing through the membrane, the permeating stream entered the vacuum chamber integrated into a Hi-Cube ECO 300 pumping station manufactured by Pfeiffer (Asslar, Germany). This pumping system provided continuous evacuation of gases from the permeate side of the membrane module. The permeate pressure was monitored by a Pfeiffer MPT200 pressure transducer (Germany). To protect the vacuum equipment from sudden pressure increases caused, for example, by membrane failure, a Pfeiffer DVC 025 PX solenoid-actuated diaphragm valve (Germany) was positioned between the membrane module and the pumping station. The permeate stream was then directed to a QMG 250 M2 quadrupole mass spectrometer produced by Pfeiffer (Germany), which was connected to a separate Pfeiffer Hi-Cube 80 pumping station (Germany). The pressure in the mass spectrometer chamber was monitored using a second pressure transmitter [52].

Before starting a measurement, the membrane module was conditioned by helium purging at 50–150 cm^3^ min^−1^. At the same time, the mixing tank was charged either with single gases or with multicomponent streams in order to generate the desired feed composition; the overall flow rate did not exceed 750 cm^3^ min^−1^. In experiments not involving argon permeation, argon was additionally fed to the vacuum-side distribution line at 4 cm^3^ min^−1^ to serve as an internal reference. The helium flushing step ensured complete removal of atmospheric air and residual gases left in the setup after preceding tests.

Gas transport through the membrane was followed by quadrupole mass spectrometry with a temporal resolution of 1 ms for raw signal acquisition. After the purge stage, the switching valve redirected the gas flow from the mixing tank to the membrane feed side with an 8 ms response delay. Operational parameters, including pressure in the membrane module and gas flow rates, were logged in FlowPlot. Measurements of sub-membrane pressure and mass-spectral signals were performed using PV MassSpec and PV TurboViewer. As a result, all experimental signals were registered online, allowing point-by-point determination of permeance using the equations given below.

Permeability coefficient (*P*) is determined in accordance with

(1) P=Ji l∆p A,

In Equation (1), *J_i_* is component *i* volumetric flow rate, cm^3^ min^−1^; *Δp* is component *i* partial pressure difference across the membrane, cmHg; and the membrane area is *A*, cm^2^. The permeability values were calculated only after stabilization of the permeate signal and flow parameters, i.e., after steady-state conditions had been reached. Under the applied conditions, this stabilization occurred within a relatively short time.

The permeability coefficient further represented in Barrer units in accordance with the following equation:

(2) $$1\;Barrer=1\times 10^{10}\;{cm}^{3}\;cm\;{cm}^{-2}\;s^{-1}\;{cmHg}^{-1}.$$

Diffusion coefficient (*D*) was obtained through the time-lag (*θ*) technique [53,54], where

(3) D=l26 θ.

The diffusion coefficient is determined using the transient region of the permeability kinetic curve. For small molecules, such as hydrogen and helium, this region is typically extremely short and because of that a number of thicker films were used to obtain averaged value.

The transient behavior was specifically relevant for the determination of diffusion coefficients of fast penetrants such as H_2_ and He; therefore, thicker films were used to increase the measurable time lag.

After the permeability and diffusion coefficients were obtained, it was possible to calculate the sorption coefficient (*S*), which is equal to

(4) S=PD.

In case of asymmetric hollow fibers, the permeance (*Q*) unit is used to characterize their mass transfer ability:

(5) Q = Ji∆p A.

Membrane selectivity is calculated according to the following equation, where permeance or permeability coefficients of two different gases may be used:

(6) α= QAQB,

The software of the mass spectrometer allows transformation of the signal of each component into the value of its partial pressure. Thus, the permeate volumetric flow rate may be estimated by the formula

(7) $$\frac{J_{i}}{J_{Ar}}=\frac{p_{i}}{p_{Ar}},$$

where *J_Ar_* is the volumetric flow rate of argon, cm^3^ min^−1^; pi is the partial pressure of component *i* in the permeate, cmHg; and *p_Ar_* is the partial pressure of argon in the permeate, cmHg.

In order to determine each component flux through the membrane, a calibration procedure was implemented in full accordance with the method reported by Fraga et al. [55]. The specific *m*/*z* value was chosen for each component of the gas mixture:

H_2_—*m*/*z* = 2;

N_2_—*m*/*z* = 28;

O_2_—*m*/*z* = 32;

CH_4_—*m*/*z* = 15;

CO_2_—*m*/*z* = 44;

H_2_S—*m*/*z* = 34.

The H_2_ (*m*/*z* = 2), CO_2_ (*m*/*z* = 44), CH_4_ (*m*/*z* = 15) and H_2_S (*m*/*z* = 34) fluxes were determined using Equation (7) and adjusted for detector relative sensitivity in full accordance with the above-mentioned study [55]. MS detector response to oxygen (*m*/*z* = 32) and nitrogen (*m*/*z* = 28) was also influenced by CO_2_ *m*/*z* = 28 fragments and H_2_S *m*/*z* = 32 fragments. Because of this, the share of each component in the overall detector signal was investigated through a preliminary study for binary (CO_2_/N_2_ and H_2_S/O_2_) systems obtained via dynamic flux mixing and corresponding coefficients were applied.

The error does not exceed ±2.2% of the measured value. Each fiber batch was characterized through the repetitive measurement of 3 to 5 of lab-scale modules with further averaging of the measured values. The deviation from sample to sample did not exceed 3.7%.

### 2.5. Biogas Upgrading Test

**Figure 3:**
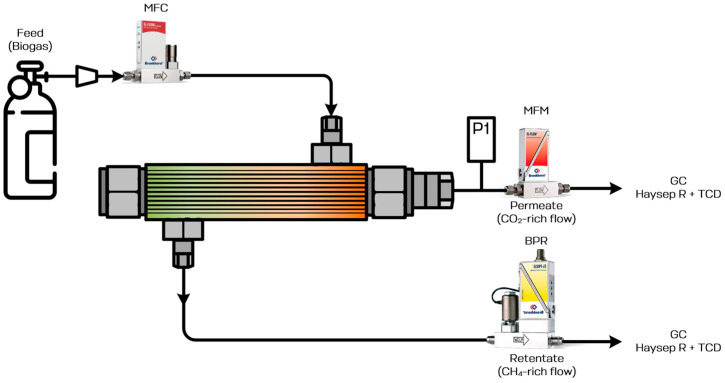
Schematic diagram of the experimental unit for biogas upgrading test.

The biogas upgrading process was studied using the experimental unit, which is schematically shown in Figure 3. The experimental unit includes membrane module (details are given below in Section 3.4), gas cylinder with pressurized biogas, mass flow controller (MFC), mass flow meter (MFM), back-pressure regulator (BPR) and pressure transmitter (P_1_).

The pressurized biogas from the gas cylinder was supplied to the membrane module inlet through the pressure regulator and MFC FG-201CV by Bronkhorst (The Netherlands) and separated into two flows: CH_4_-rich flow (retentate) and CO_2_ + H_2_S-rich flow (permeate). The pressure above the membrane was constant and equal to 0.5 MPa. It was maintained using a BPR P-702CM by Bronkhorst (The Netherlands). The permeate flow rate was measured using a MFM F-201CM by Bronkhorst (The Netherlands) and the pressure was monitored using a pressure transmitter S-10 by Wika (Lawrenceville, GA, USA). Flow composition was determined using the GC TCD [56] technique with a GC-1000 by Chromos (Dzerzhinsk, Russia) under a constant carrier gas flow rate (He 99.995%) of 20 cm^3^ min^−1^ using a packed column Haysep R at 120 °C. The fed flow was varied from 0.01 to 1 dm^3^ min^−1^. The permeate-side pressure was determined by experimental conditions and was in the range of 0.001 to 0.02 MPa.

## 3. Results and Discussion

### 3.1. PEI Film Mass Transfer Properties

First, the obtained PEI films were examined for their mass transfer characteristics with regard to single and mixed gases. Table 1 contains data obtained for neat PEI (ultem-1000) polymer films (permeability, diffusion and sorption coefficients), which may be used for further prediction of their behavior under more complex conditions. As is seen, the obtained data reveal a clear separation between diffusion-controlled and sorption-controlled transport contributions. Permeability follows the order H_2_ (8.0 Barrer) > H_2_S (1.92) ~ CO_2_ (1.76) ≫ O_2_ (0.39) > N_2_ (0.056) > CH_4_ (0.034), whereas diffusivity decreases as H_2_ (36.36 × 10^−8^ cm^2^ s^−1^) ≫ O_2_ (1.44 × 10^−8^) > H_2_S (0.39 × 10^−8^) ~ CO_2_ (0.37 × 10^−8^) > N_2_ (0.33 × 10^−8^) > CH_4_ (0.10 × 10^−8^ cm^2^ s^−1^). In contrast, solubility is highest for the acid gases, H_2_S (49.20 × 10^−3^ cm^3^ cm^−3^ cmHg^−1^) ~ CO_2_ (47.57 × 10^−3^), and much lower for H_2_, O_2_, N_2_, and CH_4_. In the context of the solution–diffusion mechanism, where *P* = *D* × *S*, these data indicate that hydrogen permeates rapidly primarily because of its exceptionally high diffusivity, whereas the relatively high permeabilities of CO_2_ and H_2_S originate mainly from their very high solubility in the PEI matrix rather than from fast diffusion. This interpretation is fully consistent with the standard solution–diffusion mechanism used for most gas separation membranes, and the measured $P_{H_{2}}$ and $P_{{CO}_{2}}$ are also close to literature values reported for neat Ultem-based PEI films [57].

At the same time, the high sorption affinity of CO_2_ and H_2_S toward the glassy PEI matrix suggests that these high-permeable components should also be regarded as the most likely plasticizing agents for this polymer. Therefore, although their transport under the present conditions is still adequately described within the solution–diffusion framework, the behavior of these acid gases may deviate from that expected for an ideally rigid matrix at elevated pressures or activities due to penetrant-induced polymer relaxation. In this sense, the relatively high permeability of CO_2_ and H_2_S reflects not only their strong sorption contribution, but also their greater ability to condition the PEI matrix compared with weakly sorbing gases.

When comparing these transport data with the kinetic diameters of the penetrants, an overall inverse size–diffusivity relationship is observed, but it is clearly not strictly linear. The smallest molecule, H_2_ (289 pm), exhibits by far the highest diffusivity, while the largest one, CH_4_ (380 pm), shows the lowest diffusivity, which is consistent with a size-sieving effect in the glassy PEI matrix. However, important deviations from a simple diameter-controlled trend are also evident. In particular, O_2_ (346 pm) diffuses substantially faster than CO_2_ (330 pm), even though CO_2_ is slightly smaller. Likewise, H_2_S (360 pm) and N_2_ (364 pm) have very similar kinetic diameters, but H_2_S shows a somewhat higher diffusivity and an enormously higher solubility. These deviations indicate that penetrant size is only a first-order descriptor of diffusion in PEI. Specific penetrant–polymer interactions, molecular condensability, and the resulting transient immobilization effects must also be considered. Thus, for CO_2_ and H_2_S, the transport behavior is governed by a combination of moderate diffusivity and very strong sorption, whereas for H_2_ the dominant contribution is fast molecular diffusion through the available free volume of the polymer.

Published studies on Ultem-based PEI membranes indicate that diffusivity in H_2_-containing systems has been evaluated from sorption or transient permeation measurements, although numerical values are often omitted. In patent documents, transport performance is typically reported in terms of permeability, permeance, and selectivity, whereas explicit diffusion coefficients are usually not provided. This tendency is consistent with the experimental difficulty of determining membrane diffusion coefficient with respect to hydrogen, since it often produces extremely short time lags in dense glassy polymers. Therefore, the present hydrogen diffusivity value is of particular interest, although its interpretation should account for the note that it was obtained as an average value for a number of thick-film samples.

Overall, the obtained results confirm that gas transport in dense PEI (Ultem-1000) cannot be explained solely on the basis of kinetic diameter. Instead, the transport behavior arises from the combined effect of molecular size and thermodynamic affinity toward the polymer. Hydrogen behaves as a weakly sorbing and highly mobile penetrant, and its transport is therefore diffusion-dominated. In contrast, CO_2_ and H_2_S exhibit only moderate diffusivities but very high solubilities, which makes their permeability predominantly solubility-controlled. Thus, PEI demonstrates a characteristic combination of strong resistance to larger penetrants such as CH_4_ and enhanced transport of hydrogen and acid gases, which supports its suitability for membrane applications involving hydrogen recovery and selective removal of acidic components.

After the PEI film mass transfer properties were obtained for the single gases, the mixed-gas permeation tests were performed. Membrane permeability, diffusion and sorption coefficients for each component of the six-component gas mixture are given in Table 2. In addition to the mass transfer properties determined initially for the films exposed to the gas mixture for the first time, the same properties were studied for the films exposed to the same mixture for 24 h. This was done in order to study the changes in the characteristics under the influence of plasticizing components present in the gas mixture (CO_2_ and H_2_S).

Comparison of Table 1 and Table 2 shows that, in the initial state, the mixed-gas transport coefficients are generally lower than those obtained in the pure-gas experiments. For CH_4_, CO_2_, H_2_S, H_2_, and N_2_, the permeability decreases from 0.034, 1.76, 1.92, 8.0, and 0.056 Barrer for the pure gases to 0.031, 1.52, 1.65, 7.3, and 0.048 Barrer, respectively, in the fresh-film mixed-gas experiment. In contrast, the corresponding diffusion coefficients remain nearly unchanged, with only minor variations: 0.10 to 0.10 for CH_4_, 0.37 to 0.36 for CO_2_, 0.39 to 0.38 for H_2_S, 36.36 to 36.20 for H_2_, and 0.33 to 0.33 × 10^−8^ cm^2^ s^−1^ for N_2_. Therefore, the initial reduction in permeability is mainly associated with a decrease in sorption rather than with any pronounced suppression of diffusion. This trend is especially evident for CO_2_ and H_2_S, whose sorption coefficients decrease from 47.57 and 49.20 to 41.99 and 43.19 × 10^3^ cm^3^ cm^−3^ cmHg^−1^, respectively. Thus, under mixed-gas conditions, competitive sorption between penetrants appears to reduce the apparent solubility of the more condensable components in the fresh PEI film.

After 24 h exposure, the transport properties of all gases increase relative to the initial mixed-gas state. The permeability rises to 0.038 Barrer for CH_4_, 1.72 for CO_2_, 1.97 for H_2_S, 8.5 for H_2_, 0.055 for N_2_, and 0.39 for O_2_. At the same time, the diffusion coefficients either remain almost constant or increase only slightly, reaching 0.11, 0.38, 0.41, 37.00, 0.33, and 1.45 × 10^−8^ cm^2^ s^−1^ for CH_4_, CO_2_, H_2_S, H_2_, N_2_, and O_2_, respectively. The increase in permeability is therefore again governed predominantly by the sorption term. For example, the sorption coefficient of CO_2_ rises from 41.99 to 44.79 × 10^3^ cm^3^ cm^−3^ cmHg^−1^, while that of H_2_S increases from 43.19 to 47.70 × 10^3^ cm^3^ cm^−3^ cmHg^−1^. A similar tendency is observed for CH_4_, H_2_, and N_2_. These results indicate that prolonged contact with the multicomponent mixture leads to conditioning of the polymer matrix and to a moderate increase in its sorption capacity, which is consistent with the action of CO_2_ and H_2_S as penetrants capable of loosening chain packing and increasing the accessible free volume of the glassy polymer.

A noticeable feature of the mixed-gas data is the deviation of hydrogen permeability from the pure-gas value. The initial hydrogen permeability (7.3 Barrer) is lower than that measured for the pure gas (8.0 Barrer), which can be attributed primarily to the significantly reduced partial pressure of hydrogen in the mixture (1.92 mol.%). Although permeability is often assumed to be independent of pressure, this assumption is strictly valid for ideal single-gas conditions, whereas under mixed-gas conditions the effective transport may be influenced by both reduced driving force and the simultaneous permeation of other species. In contrast to condensable gases, hydrogen transport in PEI is predominantly diffusion-controlled, and therefore competitive sorption effects are not expected to play a major role.

After 24 h exposure, the hydrogen permeability increases to 8.5 Barrer, exceeding the pure-gas value. This behavior is consistent with a mild conditioning or moderate plasticization of the polymer matrix induced by strongly sorbing components such as CO_2_ and H_2_S. The fact that permeability increases for all gases supports the interpretation that the polymer free volume is slightly increased, rather than indicating a measurement artifact. At the same time, the magnitude of this increase remains moderate, suggesting that the polymer structure is not severely disrupted but undergoes limited relaxation under prolonged exposure to the gas mixture.

However, the obtained data do not permit an unambiguous assessment of whether this conditioning effect is fully reversible or persists during long-term operation. Since the permeability increase after 24 h is associated predominantly with enhanced sorption, whereas the diffusion coefficients show only minor changes, the observed behavior is better described as moderate penetrant-induced conditioning of the glassy PEI matrix rather than as severe irreversible plasticization. This distinction is important because it implies preservation of the main diffusion-controlled selectivity features of the polymer despite the increase in sorption capacity. Nevertheless, dedicated desorption, recovery, and long-term aging experiments are necessary to establish whether the transport parameters return to their initial values after removal of the gas mixture or whether partial structural relaxation remains. Thus, under the present conditions, the results point to mild conditioning of PEI by the multicomponent gas mixture, while the reversibility and long-term stability of this effect remain open questions for further investigation.

The most pronounced effect is observed for oxygen. In the initial mixed-gas measurement, the O_2_ permeability is only 0.035 Barrer and the sorption coefficient is 0.24 × 10^3^ cm^3^ cm^−3^ cmHg^−1^, whereas after 24 h exposure these parameters increase to 0.39 Barrer and 2.69 × 10^3^ cm^3^ cm^−3^ cmHg^−1^, respectively, i.e., to values very close to those obtained in the pure-gas experiment. At the same time, the O_2_ diffusion coefficient changes only marginally, from 1.43 to 1.45 × 10^−8^ cm^2^ s^−1^. This behavior indicates that the large difference between the initial and exposed states is almost entirely sorption-driven. In other words, the initially fresh film exhibits strongly suppressed apparent O_2_ solubility under mixed-gas conditions, whereas after prolonged exposure the membrane reaches a conditioned state in which oxygen transport approaches its pure-gas behavior.

Overall, the mixed-gas results demonstrate that the transport behavior of the PEI film is controlled not only by penetrant size but also by competitive sorption and mixture-induced conditioning of the polymer matrix. Hydrogen remains the fastest penetrant because of its very high diffusivity, while CO_2_ and H_2_S retain high permeability primarily due to their high sorption affinity toward PEI. Methane and nitrogen remain the least permeable components, reflecting their low diffusivity and weak sorption. The fact that, after 24 h exposure, the mixed-gas transport coefficients become close to or even slightly exceed the pure-gas values suggests that the effect of the CO_2_/H_2_S-containing mixture is manifested mainly as moderate reversible conditioning or mild plasticization of the PEI film rather than as severe irreversible structural damage.

### 3.2. PEI Hollow-Fiber Characterization

After the PEI film mass transfer properties were studied with respect to single and mixed gases, it was possible to determine the intrinsic selectivity values for gas pairs of interest (O_2_/N_2_, H_2_/CH_4_, CO_2_/CH_4_, H_2_S/CH_4_ and CO_2_/N_2_), which may be used as a reference value when studying hollow fibers. The hollow fibers were studied in the same way as PEI films in the case of mixed gases and the experimental data refer to the exposed state. Table 3 shows the experimental data on selectivity values of PEI films after the 24 h exposure to quasi-real biogas. Table 4 shows accumulated data for different PEI hollow fibers obtained from NMP- and DMF-based dope solutions at 25 and 50 °C.

The mixed-gas selectivity of the dense PEI film provides the intrinsic separation benchmark for the hollow-fiber membranes. As shown in Table 3, the film exhibits high selectivity toward the light or strongly sorbing gases of the mixture, with values of 223.7 (H_2_/CH_4_), 51.8 (H_2_S/CH_4_), 45.3 (CO_2_/CH_4_) and 31.3 (CO_2_/N_2_); meanwhile, the O_2_/N_2_ selectivity remains 7.1. These values are consistent with the transport characteristics previously obtained for the PEI film, where hydrogen transport is predominantly diffusion-controlled, whereas CO_2_ and H_2_S benefit from their high sorption affinity toward the polymer matrix. Therefore, the film data define the upper selectivity level that can be expected from asymmetric hollow fibers if the outer skin layer is dense and essentially defect-free.

For the hollow fibers spun from the NMP-based dope, the most selective one was obtained at an air gap of 16 cm (Table 5). Under these conditions, the mixed-gas selectivity remained close to that of the dense film. At the same time, the permeance remained low, e.g., for H_2_ 51.1–61.1 GPU and for CO_2_ 10.1–13.1 GPU. Thus, the NMP-spun fibers at 16 cm reproduced the intrinsic selectivity of the film reasonably well, indicating formation of a relatively thick and dense selective skin layer. Its thickness may be evaluated from permeability coefficient (*P*) of oxygen using hollow-fiber permeance for the same gas. Dividing the dense-film permeability coefficient value (for 24 h exposed sample) by the permeance value (50 °C and 16 cm air gap sample) yields 140 nm.

When the air gap was reduced to 12 cm, all gas permeances increased noticeably, but this gain was accompanied by a systematic decrease in selectivity. For example, at 50 °C, H_2_ permeance increased to 102.7 GPU, whereas α(H_2_/CH_4_) decreased to 197.4. A further decrease in the air gap to 8 cm led to a sharp increase in permeance, especially for H_2_ (278–311.6 GPU), CO_2_ (27.1–30.3 GPU) and CH_4_ (7.8–8.6 GPU), but the selectivity collapsed to nearly nonselective levels. This behavior strongly suggests the formation of defective or imperfect selective layers at the shortest air gap.

A similar but more pronounced trend was observed for the hollow fibers prepared from the DMF-based dope. At an air gap of 16 cm, the fibers already exhibited substantially higher permeance than their NMP-based counterparts, for example, *Q*(H_2_) = 127.6–131.7 GPU and *Q*(CO_2_) = 25.3–26.1 GPU, while the selectivity (Table 6) was somewhat lower than that of the film and also lower than that of the best NMP-spun fibers. At 50 °C, selectivity for pair H_2_/CH_4_ = 209.2 and CO_2_/CH_4_ = 41.5; at 25 °C these values further decreased to 156.8 and 31.1, respectively. Reducing the air gap to 12 cm caused a drastic loss of selectivity despite very high permeance. At 8 cm, meaningful selectivity values were no longer obtained for the DMF-based fibers due to a lack of stability in the membrane-forming process—specifically, frequent ruptures at 8 cm air gap at 25 °C. An air gap of 8 cm at 50 °C allowed for spinning extremely high-permeable fiber; nevertheless, permeance for hydrogen, carbon dioxide and hydrogen sulfide was too high to precisely determine via the above-mentioned technique.

**Figure 4:**
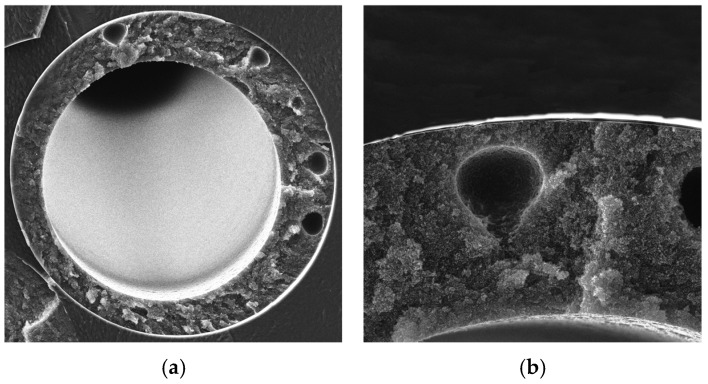

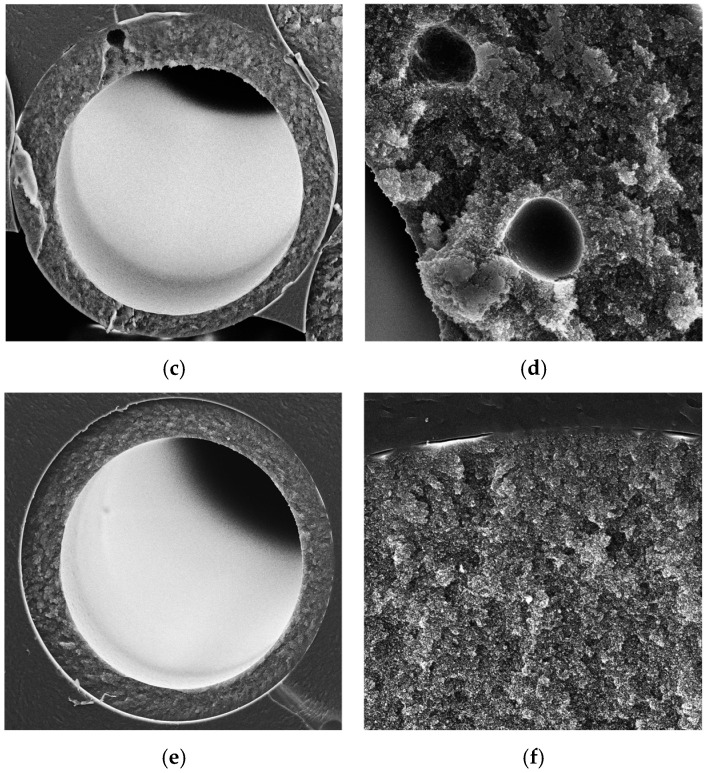
Cross-section SEM microphotographs of obtained fibers. (**a**,**b**)—DMF-based fiber obtained at 50 °C and 8 cm air gap; (**c**,**d**)—DMF-based fiber obtained at 50 °C and 12 cm air gap; (**e**,**f**)—NMP-based fiber obtained at 50 °C and 16 cm air gap.

A direct comparison of the two solvents shows that NMP provides a much better permeance–selectivity trade-off than DMF for asymmetric PEI hollow fibers. The NMP-based fibers, especially at 16 cm air gap, remain close to the film benchmark, whereas the DMF-based fibers shift more rapidly toward high-permeance and low-selective behavior. This difference is fully consistent with the available literature on PEI solution thermodynamics and hollow-fiber formation [58]. As is known, NMP is the stronger solvent for PEI; the solvent power decreases in the order NMP > DMAc > DMF. Binary-solvent studies on PEI hollow fibers have shown that solvent composition can shift the morphology from macrovoidal to sponge-like structures, confirming the high sensitivity of PEI hollow-fiber formation to the thermodynamic state of the dope. Likewise, classical gas separation studies on PEI hollow fibers identified NMP, at a suitable air gap, as a solvent capable of producing high-selectivity skins. To study this effect, SEM microphotographs of the cross-section were obtained for several samples (Figure 4).

Morphological features of PEI hollow fibers. The SEM micrographs reveal a pronounced effect of both solvent nature and air gap distance on the cross-sectional morphology of the PEI hollow fibers. The fibers obtained from DMF-based dope at 50 °C and air gap of 8 cm exhibit the most open and structurally heterogeneous architecture among the three membranes. The cross-section contains several large rounded macrovoids embedded in a highly porous matrix, while the supporting region appears relatively loose and insufficiently compact. Such a morphology is characteristic of rapid liquid–liquid demixing during phase inversion and is consistent with the use of DMF, which, according to the discussion above, behaves as a weaker solvent for PEI than NMP and therefore promotes earlier precipitation of the polymer. In addition, the short air gap of 8 cm provides limited time for stabilization of the nascent outer layer prior to immersion in the coagulation bath. As a result, the membrane develops a highly permeable but poorly selective structure, which agrees well with the mixed-gas permeation data, where this sample showed extremely high permeance and nearly collapsed selectivity.

A transition toward a more ordered structure is observed for the fibers obtained from DMF-based dope at 50 °C and air gap of 12 cm. In this case, macrovoids are still clearly present, but they are fewer and more isolated, while the surrounding polymer matrix appears more continuous than in previously discussed samples. Thus, increasing the air gap from 8 to 12 cm partially suppresses the most severe structural defects and allows a more stable selective layer to form, although the membrane remains distinctly macrovoid-containing. This intermediate morphology is fully consistent with its transport behavior: compared with 8 cm air gap, the higher air gap (12 cm) provides relatively high gas permeance, but its selectivity is noticeably improved. Therefore, the fiber structure obtained from the DMF-based dope at 50 °C and air gap of 12 cm may be regarded as a compromise morphology, in which the porous substructure still provides high flux, whereas the outer layer becomes sufficiently continuous to allow further improvement by silicone post-treatment.

In contrast, fibers obtained from NMP-based dope at 50 °C and air gap of 16 cm demonstrate a much more homogeneous and compact sponge-like structure. No large macrovoids are visible in the magnified image, and the membrane wall appears substantially more uniform over the entire thickness. The outer contour is smooth, and the selective region appears continuous, indicating a more controlled phase inversion process. This morphology is in good agreement with the stronger solvating ability of NMP toward PEI and with the longer air gap of 16 cm, both of which favor delayed demixing and partial structural fixation of the nascent fiber before coagulation. As a consequence, NMP-based fibers (50 °C and 16 cm air gap) exhibit the structure expected for an integrally selective membrane, which correlates well with the experimentally observed low permeance and selectivity values closest to those of the dense PEI film.

Overall, the SEM observations clearly support the previously established structure–property relationships for the studied PEI hollow fibers. A shift from 8 to 12 cm (DMF-based fibers) and to 16 cm (NMP-based fibers) corresponds to a transition from a highly porous macrovoid-rich morphology to a more homogeneous sponge-like structure with a better-defined selective layer. This morphological evolution directly explains the decrease in permeance and the simultaneous increase in mixed-gas selectivity observed in the transport experiments. In other words, the presence of large macrovoids and loose substructure is associated with fast but partially nonselective gas transport, whereas suppression of macrovoid formation and development of a more uniform sponge-like matrix lead to behavior closer to the intrinsic selectivity of the PEI material. Accordingly, the SEM analysis confirms that NMP with a longer air gap is more favorable for fabrication of intrinsically selective PEI hollow fibers, while DMF-based spinning promotes more open and defect-prone structures, especially at short air gaps.

The observed influence of air gap can also be rationalized in terms of solvent evaporation and nascent-fiber stabilization before immersion in the coagulation bath. A longer air gap favors partial solvent loss and a greater degree of structural fixation of the outer layer, which helps preserve a dense selective skin. In contrast, shortening the air gap increases permeance but progressively sacrifices selectivity, indicating that the skin becomes thinner, less uniform, or partially defective. This effect is especially strong for the DMF-based dope, which appears to be more demanding to with respect to phase inversion conditions. From a practical viewpoint, these results indicate that NMP is the preferred solvent for preparing integrally selective PEI hollow fibers for gas separation, whereas DMF is more suitable when a highly porous or highly permeable structure is desired.

Overall, the hollow-fiber data show that the intrinsic mixed-gas selectivity of PEI can be translated into hollow-fiber form only under sufficiently mild spinning conditions that allow formation of a continuous defect-free outer skin. Among the investigated conditions, the best compromise between permeance and selectivity was achieved for the NMP-based dope at 16 cm air gap, while decreasing the air gap and especially replacing NMP with DMF shifted the membrane structure toward a more permeable but markedly less selective state.

### 3.3. Hollow-Fiber Treatment

After the differently obtained batches of hollow fibers were spun, the silicone treatment procedure was applied to the most permeable ones and the mass transfer properties were studied again for comparison with untreated fibers and among treated batches.

For this purpose, three samples were chosen, obtained from NMP-based dope solution spun under 8 cm air gap at 25 °C (NMP_8_25) and DMF-based dope solution spun under 12 cm air gap at 25 and 50 °C (DMF_12_25 and DMF_12_50).

Treatment was performed using Sylgard 184 silicone coating solution in n-heptane (1 and 3 wt.%). Stainless steel housings containing 50 hollow fibers each were filled with silicone solution and placed on a vibrational table for 8 h. Afterwards, excess solution was removed from the modules and they were dried at 50 °C under continuous nitrogen flow supplied on the lumen side of the fibers. For further comparison, treated samples were marked with Trtd prefix. The mass transfer properties of treated samples are given in Table 7 and Table 8 for 1 and 3 wt.% silicone solutions respectively.

As is seen from Table 7 and Table 8, the applied silicone treatment noticeably affected the mixed-gas transport behavior of the PEI hollow fibers and, in general, shifted their separation performance toward the intrinsic behavior of the dense PEI film. Since direct comparison of film permeability coefficient and hollow-fiber permeance values is not straightforward because of the different geometry and insufficiently reliable (assessed based on oxygen permeance) effective selective-layer thickness, the most meaningful criterion is the selectivity pattern. For the dense PEI film, the characteristic selectivity values for CO_2_/CH_4_, H_2_S/CH_4_ and CO_2_/N_2_ were 45.3, 51.8 and 31.3 respectively, which can be regarded as the intrinsic separation benchmark of the polymer. The untreated hollow fibers, especially those prepared under less favorable spinning conditions, exhibited substantially lower selectivities, indicating the presence of nonselective transport pathways. In this context, silicone coating was expected to suppress surface defects (micro pinholes) and to reduce the contribution of nonselective leakage, thereby increasing the acid gas selectivity of the fibers.

The effect of the 1 wt.% silicone solution in n-heptane was positive but rather limited. For the NMP_8_25 sample, the treatment increased CO_2_/CH_4_ selectivity from approximately 3.5 for the untreated sample to 4.6 and CO_2_/N_2_ from 3.4 to 4.5, while the permeance of CH_4_ and N_2_ decreased only moderately. A similar tendency was observed for DMF_12_25, for which CO_2_/CH_4_ selectivity increased from about 5.6 to 6.3 and for CO_2_/N_2_ pair from 5.1 to 6.0. For DMF_12_50, the 1 wt.% treatment led only to a marginal increase in CO_2_/CH_4_ selectivity from 6.6 to 6.9, although CO_2_/N_2_ selectivity rose somewhat more noticeably from 4.9 to 6.6. Overall, the 1 wt.% coating did not bring the fibers close to the dense-film selectivity level. This suggests that such a low silicone concentration is insufficient for complete sealing of surface imperfections and interfacial defects in the asymmetric PEI hollow fibers.

A much more pronounced effect was obtained after treatment with the 3 wt.% silicone solution. In all three cases, CH_4_ and N_2_ permeance decreased far more strongly than those values for CO_2_ and H_2_S, which resulted in a substantial increase in selectivity. For NMP_8_25, membrane permeance with regard to CH_4_ decreased from 8.6 to 1.8 GPU and to N_2_ from 8.8 to 1.9 GPU, whereas CO_2_ remained relatively high at 24.6 GPU. As a result, CO_2_/CH_4_ increased to 13.8 and CO_2_/N_2_ to 13.1. For DMF_12_25, the 3 wt.% coating reduced membrane permeance to CH_4_ from 10.2 to 1.9 GPU and N_2_ permeance decreased from 11.32 to 2.1 GPU, while carbon dioxide permeance remained at 27.7 GPU; correspondingly, selectivity to gas pairs CO_2_/CH_4_ and CO_2_/N_2_ increased to 14.6 and 13.2, respectively. The most remarkable result was obtained for DMF_12_50, where the 3 wt.% coating yielded membrane permeance to CO_2_, H_2_S, CH_4_ and N_2_ at 39.4, 47.12, 1.76 and 1.95. This provides the following selectivity values for CO_2_/CH_4_, H_2_S/CH_4_ and CO_2_/N_2_ of 22.4, 26.8 and 20.2 respectively, which are the highest values among all treated samples. These results clearly show that increasing the silicone concentration from 1 to 3 wt.% is much more effective for suppressing nonselective transport.

This behavior is consistent with the intrinsic transport properties of PEI established earlier for the dense film. In the dense polymer, hydrogen has the highest diffusivity because of its smallest kinetic diameter, whereas CO_2_ and H_2_S exhibit high permeability primarily because of their strong sorption in the polymer matrix. Therefore, in the coated fibers the preservation of relatively high acid gases permeance, accompanied by a strong decrease in membrane permeance with respect to methane and nitrogen, indicates that silicone treatment indeed suppresses nonselective flow channels and shifts the transport mechanism closer to solution–diffusion-controlled permeation through the polymer-selective layer. The fact that the order of permeance remains H_2_ ≫ H_2_S ~ CO_2_ > O_2_ > N_2_ ~ CH_4_ further supports this interpretation.

At the same time, comparison with the untreated fibers shows that silicone coating is not universally advantageous for every morphology. Some untreated fibers produced under more favorable spinning conditions already exhibited selectivities much closer to the dense-film benchmark. For example, untreated NMP fibers with a 16 cm air gap showed very high calculated selectivities. These values are closer to the dense-film selectivity than those of the silicone-treated high-flux fibers. However, this higher selectivity was achieved at markedly higher permeance values. For instance, the highly selective untreated NMP_16 samples exhibited CO_2_ permeance of only 10.1–13.1 GPU, whereas the DMF_12_50 fiber treated with 3 wt.% silicone retained a much higher permeance of 39.4 GPU together with a substantial increase in selectivity. Thus, silicone post-treatment is especially useful for initially high-flux but poorly selective fibers, because it improves the selectivity/permeance balance rather than simply maximizing selectivity alone.

From the standpoint of biogas upgrading, the key target is preferential removal of acid gases with minimum methane loss. Based on this criteria, the DMF_12_50 fiber treated with 3 wt.% silicone in n-heptane appears to be the most promising option among the coated samples. It combines the highest CO_2_/CH_4_ and H_2_S/CH_4_ selectivity values among the treated fibers, while maintaining relatively high permeances. In contrast, the 1 wt.% silicone treatment is clearly insufficient to achieve a comparable improvement in separation performance. It should also be noted that, although the coating strongly reduces N_2_ permeance, the PEI fibers remain primarily acid-gas-selective rather than truly nitrogen-selective membranes; therefore, the main practical benefit of the treatment lies in enhanced removal of acid gases rather than in deep denitrogenation. Overall, the results demonstrate that a 3 wt.% silicone coating is a viable post-treatment strategy for converting high-permeance but defect-prone PEI hollow fibers into more selective membranes approaching the intrinsic separation behavior of the dense PEI film.

### 3.4. Biogas Upgrading

After the comprehensive study of the obtained hollow-fiber batches was carried out the lab-scale membrane module containing ~500 fibers was assembled and studied during the separation of the quasi-real biogas mixture (methane, carbon dioxide, hydrogen sulfide, hydrogen, nitrogen, and oxygen, with the following molar composition: 68.3/27.13/0.56/1.92/0.97/1.12 mol.%). The effective length of the module was 250 mm with inner diameter of 17.2 mm (3/4” stainless steel tube) resulting in effective area of 0.216 m^2^.

**Figure 5:**
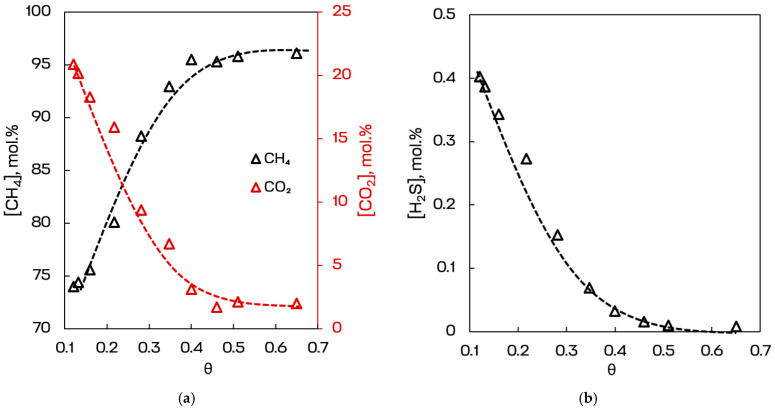
Methane, carbon dioxide (**a**) and hydrogen sulfide (**b**) concentrations in the retentate flow plotted versus stage-cut of the membrane separation process.

During this experiment the ability of the membrane to separate biogas was evaluated through the retentate CH_4_, CO_2_ and H_2_S concentrations plotted against stage-cut, which was varied via feed flow rate. The results are given in Figure 5.

The stage-cut dependencies obtained for the membrane module comprising 500 PEI hollow fibers of the DMF_12_50 type clearly demonstrate the preferential permeation of acid gases and the corresponding enrichment of methane in the retentate during separation of the six-component biogas model mixture. As the stage-cut increased from approximately 0.1 to 0.65, the methane concentration in the retentate rose monotonically from about 74 to 96 mol.%, whereas the carbon dioxide concentration decreased from about 21 to nearly 2 mol.%. Thus, the membrane module provided a pronounced upgrading effect over the whole investigated operating window. The shape of both curves indicates that the strongest change in retentate composition occurred in the intermediate stage-cut range, while at values above 0.45 the system approached a plateau, with only minor additional methane enrichment upon further increase in stage-cut.

The H_2_S profile shows an even more pronounced removal effect. The hydrogen sulfide concentration in the retentate decreased from about 0.39–0.40 mol.% at low stage-cut to values close to zero at stage-cut values of 0.45–0.5 and remained at trace level at higher stage-cut. This behavior is fully consistent with the previously established mixed-gas transport characteristics of PEI fibers, in which H_2_S and CO_2_ exhibit substantially higher permeance than CH_4_. At the same time, the much faster depletion of H_2_S compared with CO_2_ is also influenced by its significantly lower initial feed concentration. Because H_2_S is present only as a minor component, even a membrane with comparable permeance toward H_2_S and CO_2_ removes H_2_S from the retentate much more rapidly in absolute terms. As a result, the module demonstrates not only methane enrichment but also effective desulfurization, which is highly important for practical biogas treatment.

The observed concentration trends are in good agreement with the gas transport behavior discussed earlier for PEI. In this polymer, methane is the slowest of the major biogas components because of its relatively large kinetic diameter and low diffusivity, whereas CO_2_ and H_2_S are more permeable due to the combined effect of favorable sorption and higher transport rates through the selective layer. Therefore, the continuous increase in CH_4_ concentration in the retentate, accompanied by a decrease in CO_2_ and H_2_S, confirms that the membrane operates according to the expected acid-gas-selective mechanism rather than through nonselective leakage. In other words, the module preferentially transfers acid gases to the permeate side while retaining methane in the non-permeating stream.

From a process standpoint, the most practically relevant region appears to be the range of stage-cut values of approximately 0.45–0.55. In this interval, the retentate already contains about 95–96 mol.% CH_4_, the CO_2_ concentration is reduced to roughly 2–3 mol.%, and H_2_S is almost completely removed. Further increase in stage-cut above this range provides only marginal additional purification, whereas in a real process it would generally be associated with higher methane loss to the permeate and a greater process burden. Thus, the stage-cut profiles indicate the existence of a favorable compromise between methane purity and process efficiency. Such behavior is particularly important for practical membrane upgrading of biogas, where excessively high stage-cut is usually undesirable despite the somewhat higher product purity.

Overall, the obtained results demonstrate that the DMF_12_50 PEI hollow-fiber material is highly promising for biogas upgrading, primarily for simultaneous methane enrichment and removal of acid impurities. The module achieved a substantial increase in methane concentration together with removal of CO_2_ and nearly complete elimination of H_2_S, which confirms the applicability of this material for membrane-based purification of raw biogas. At the same time, the gradual flattening of the concentration curves at high stage-cut suggests that, for industrial implementation, the greatest benefit would likely be obtained in a moderate stage-cut regime or within a staged (two- or three-stage) [59] membrane process. In this sense, the studied PEI material can be regarded as an effective acid-gas-selective membrane suitable for the core upgrading step in biogas treatment, especially when the key objective is reduction in CO_2_ and H_2_S with simultaneous production of a methane-enriched retentate.

## 4. Conclusions

In this work, polyetherimide hollow-fiber membranes were developed for biogas purification, and the effects of spinning conditions, membrane morphology, silicone post-treatment, and module operating regime on separation performance were systematically evaluated. The obtained results demonstrated that the structure of the hollow fibers strongly depended on both the solvent nature and the air gap distance. Fibers prepared from DMF-based dope solutions exhibited a more open, macrovoid-rich morphology and therefore higher mixed-gas permeance, whereas NMP-based fibers formed a more homogeneous sponge-like structure with lower permeance and selectivity values closer to those of dense PEI films.

Silicone post-treatment proved to be an effective approach for reducing nonselective transport and shifting the gas separation behavior of the hollow fibers toward the intrinsic selectivity of the polymer. In particular, treatment with a 3 wt.% silicone solution in *n*-heptane was significantly more effective than treatment with a 1 wt.% solution. Among the investigated samples, the DMF_12_50 fibers treated with 3 wt.% silicone provided the most favorable balance between permeance and selectivity and therefore can be considered the most promising material for further application in biogas upgrading. These fibers combined relatively high acid gas permeance with substantially improved selectivity toward CH_4_ and N_2_, confirming the importance of post-treatment for high-flux but initially defect-prone asymmetric PEI membranes.

Testing of a membrane module containing ~500 DMF_12_50 fibers with a total area of 0.216 m^2^ confirmed the practical applicability of the developed material for separation of a six-component biogas model mixture. With increasing stage-cut, the methane concentration in the retentate increased to approximately 96 mol.%, while the carbon dioxide concentration decreased to about 2 mol.% and hydrogen sulfide was almost completely removed. These results demonstrate that PEI hollow fibers with controlled structure and optimized silicone post-treatment are highly promising for simultaneous methane enrichment, carbon dioxide removal, and deep desulfurization of biogas. Overall, the study shows that morphology control combined with defect-sealing post-treatment is a viable strategy for producing efficient acid-gas-selective PEI membranes for membrane-based biogas purification.

## Figures and Tables

**Table 1:** PEI film single-gas mass transfer characteristics.

Gas	P, Barrer	D × 10^8^, cm^2^ s^−1^	S × 10^3^, cm^3^ cm^−3^ cmHg^−1^
CH_4_	0.034	0.10	3.31
CO_2_	1.76	0.37	47.57
H_2_S	1.92	0.39	49.20
H_2_	8.0	36.36 ^a^	2.20
N_2_	0.056	0.33	1.71
O_2_	0.39	1.44	2.71

@ 110 kPa and 25 °C. ^a^—averaged value obtained for thicker samples.

**Table 2:** PEI film mixed-gas mass transfer characteristics.

Gas	P, Barrer		D × 10^8^, cm^2^ s^−1^		S × 10^3^, cm^3^ cm^−3^ cmHg^−1^	
	Initial	Exposed	Initial	Exposed	Initial	Exposed
CH_4_	0.031	0.038	0.10	0.11	3.07	3.39
CO_2_	1.52	1.72	0.36	0.38	41.99	44.79
H_2_S	1.65	1.97	0.38	0.41	43.19	47.70
H_2_	7.3	8.5	36.20	37.00	2.02	2.30
N_2_	0.048	0.055	0.33	0.33	1.47	1.68
O_2_	0.35	0.39	1.43	1.45	0.24	2.69

**Table 3:** PEI film mixed-gas selectivity (*α*) values.

	O_2_/N_2_	H_2_/CH_4_	CO_2_/CH_4_	H_2_S/CH_4_	CO_2_/N_2_
α	7.1	223.7	45.3	51.8	31.3

**Table 4:** PEI hollow-fiber mixed-gas permeance (*Q*) values.

Air Gap, cm	T, °C	Q, GPU											
		NMP Solvent						DMF Solvent					
		CH_4_	CO_2_	H_2_S	H_2_	N_2_	O_2_	CH_4_	CO_2_	H_2_S	H_2_	N_2_	O_2_
16	50	0.2	10.1	12.2	51.1	0.4	2.8	0.61	25.32	28.11	127.59	1	5.37
12		0.5	21.1	25.6	102.7	1.1	5.0	4.58	30.12	33.3	142.74	6.11	8.72
8		7.8	27.1	28.5	278.0	8.3	9.1	12.56	-	-	-	12.48	-
16	25	0.3	13.1	15.3	61.1	0.5	3.1	0.84	26.12	29.68	131.67	1.34	5.87
12		0.8	27.1	28.3	111.1	1.7	5.5	10.2	57.18	63.67	388.56	11.32	12.78
8		8.6	30.3	31.9	311.6	8.8	9.8	-	-	-	-	-	-

**Table 5:** Mixed-gas selectivity (*α*) values of PEI hollow fibers obtained from NMP-based dope.

Air Gap, cm	T, °C	α				
		O_2_/N_2_	H_2_/CH_4_	CO_2_/CH_4_	H_2_S/CH_4_	CO_2_/N_2_
16	50	6.7	212.8	42.2	50.8	24.7
12		4.5	197.4	40.6	49.2	18.9
8		1.1	35.6	3.5	3.6	3.3
16	25	6.1	191.1	40.9	47.8	25.7
12		3.3	146.1	35.7	37.3	16.4
8		1.1	36.2	3.5	3.7	3.4

**Table 6:** Mixed-gas selectivity (*α*) values of PEI hollow fibers obtained from DMF-based dope.

Air Gap, cm	T, °C	α				
		O_2_/N_2_	H_2_/CH_4_	CO_2_/CH_4_	H_2_S/CH_4_	CO_2_/N_2_
16	50	5.4	209.2	41.5	46.1	25.3
12		1.4	31.2	6.6	7.3	4.9
8		-	-	-	-	-
16	25	4.4	156.8	31.1	35.3	19.5
12		1.1	38.1	5.6	6.2	5.1
8		-	-	-	-	-

**Table 7:** Mixed-gas permeance (*Q*) values of PEI hollow fibers treated with 1 wt.% silicone solution.

	Q, GPU						α				
	CH_4_	CO_2_	H_2_S	H_2_	N_2_	O_2_	O_2_/N_2_	H_2_/CH_4_	CO_2_/CH_4_	H_2_S/CH_4_	CO_2_/N_2_
NMP_8_25	6.0	27.5	29.3	300.4	6.1	9.0	1.5	50.1	4.6	4.9	4.5
DMF_12_25	3.98	25.12	30.14	106.65	4.22	7.12	1.7	26.8	6.3	7.6	6.0
DMF_12_50	7.35	50.78	53.3	256.14	7.68	10.98	1.4	34.8	6.9	7.3	6.6

**Table 8:** Mixed-gas permeance (*Q*) values of PEI hollow fibers treated with 3 wt.% silicone solution.

	Q, GPU						α				
	CH_4_	CO_2_	H_2_S	H_2_	N_2_	O_2_	O_2_/N_2_	H_2_/CH_4_	CO_2_/CH_4_	H_2_S/CH_4_	CO_2_/N_2_
NMP_8_25	1.8	24.6	27.3	278.0	1.9	9.5	5.1	156.2	13.8	15.4	13.1
DMF_12_25	1.9	27.67	30.11	110	2.1	7.56	3.6	57.9	14.6	15.8	13.2
DMF_12_50	1.76	39.4	47.12	258.12	1.95	10.11	5.2	146.7	22.4	26.8	20.2

## Data Availability

The original contributions presented in this study are included in the article. Further inquiries can be directed to the corresponding author.
